# Refractory overactive bladder patients who chose sacral neuromodulation therapy after failed OnabotulinumtoxinA treatment: A systematic review and meta-analysis

**DOI:** 10.1371/journal.pone.0230355

**Published:** 2020-03-30

**Authors:** Guang Yang, Yong Xu, Genyi Qu, Yulong Zhang

**Affiliations:** Urology, The Affiliated Zhuzhou Hospital Xiangya Medical College CSU, ZhuZhou, China; University of Oklahoma Health Sciences Center, UNITED STATES

## Abstract

**Objective:**

To systematically review outcomes in patients with refractory overactive bladder (OAB) patients who underwent sacral neuromodulation therapy (SNM) therapy after unsuccessful onabotulinumtoxinA (BTX) therapy, and to compare outcomes with those who SNM as initial therapy.

**Methods:**

A systematic search of Cochrane Library, Pubmed and Embase databases from July 2002 to November 2019, to analyze randomized controlled trials and retrospective studies of SNM therapy after failed initial BTX therapy. Two reviewers independently screened the studies and extracted data. A quality assessment of the included literature was conducted using Newcastle-Ottawa Scale (NOS), and Stata 12.0 software was used to conduct a meta-analysis of the collected data.

**Results:**

A total of seven studies involving 319 patients were finally included. The success rate in refractory OAB patients who used SNM therapy after failed BTX therapy was 58.5%, 95% CI (0.47–0.70). There was no significant difference between refractory OAB patients who chose SNM as replacement therapy after failed BTX therapy and those who used SNM therapy as first choice [RR = 0.96, 95%CI (0.72–1.26), P = 0.735].

**Conclusion:**

OAB patients for whom an initial choice of BTX therapy ends in failure or dissatisfaction may consider switching to SNM therapy. There is no difference in outcomes between these patients and those whose first choice was SNM therapy.

## Introduction

The International Continence Society (ICS) committee has defined OAB as “a syndrome characterized by symptoms of urgency, with or without urgency incontinence, usually with increased daytime frequency and nocturia (increased night time urination). OAB includes detrusor instability and hyperreflexia, both of which are described as neurogenic detrusor overactivity (NDO)[1]. In a telephonic survey of six European countries[2], the overall prevalence of OAB among individuals 40 years and older of both genders was 16.6%. Its incidence surpassed that of Alzheimer disease and osteoporosis. An overall 6.0% prevalence rate of OAB (5.9% of males, 6.0% of females) was reported from China’s first large-scale survey of communicable diseases[3].

Traditional treatment options for OAB’s include: ① Behavioral therapy with bladder training, biofeedback, pelvic floor muscle exercise, and hypnotherapy. ② Pharmacological therapy with anticholinergics and muscarinic receptor antagonists. When these treatments become ineffective or unsuitable, injections of BTX into the detrusor muscle and SNM are the third line treatment. Both methods have their own advantages and disadvantages.

Amundsen et al.[4] reported a multicenter random trial of BTX therapy for refractory urgency urinary incontinence and found that the efficacy was higher than of SNM; however, BTX therapy often led to urinary tract infections and the need for transient self-catheterizations. Dowson et al.[5] found that at least 37% of patients stopped using BTX therapy after the first two injections, and that the most commonly reported reasons for doing so were that it was ineffective and that there were related problems caused by intermittent urinary catheters. As for refractory OAB patients who initially chose BTX therapy, but for whom it was later ineffective or they stopped using it due to adverse reactions, whether or not to continue choosing SNM therapy is a clinical issue that must be addressed. After failure of BTX therapy for refractory OAB, there is a possibility that an even more resistant form of OAB will appear[6]. This creates limitations as to the purposes for using SNM replacement therapy. In the present study, we performed a meta-analysis to address the question of whether or not patients with refractory OAB can continue to use SNM therapy after the initial choice and failure of BTX therapy.

## Materials and methods

### Inclusion and exclusion criteria

Inclusion criteria were as follows: randomized controlled trials and observational studies involving refractory OAB patients who chose SNM therapy after ending BTX therapy; urodynamic tests confirmed that the patients had detrusor overactivity; other related types of diagnoses were excluded; the control group included refractory OAB patients who initially chose SNM therapy. There were no limitations regarding gender, ethnicity, or nationality. If there were identical studies from the same research group, the most recent and most comprehensive study was chosen. Observation indexes were as follows: the success rate in patients with refractory OAB after receiving therapy, including recordings of voiding diary, quality of life (QOL), OAB symptom scores (OABSS), residual urine volume (bladder ultrasound measurement), maximum flow rate, and others. The level of improvement of the main symptoms reached or exceeded 50%, while the therapeutic efficacy met the patients’ expectations, and was regarded as successful treatment of refractory OAB. Exclusion criteria were as follows: OAB combined with other urinary diseases (including stones, tumors, urinary tract infections, and others.); non-English or duplicate publications; data reported in the literature that were incomplete, unusable, or could not be acquired by contacting the author.

### Literature retrieval strategy

**Fig 1:**
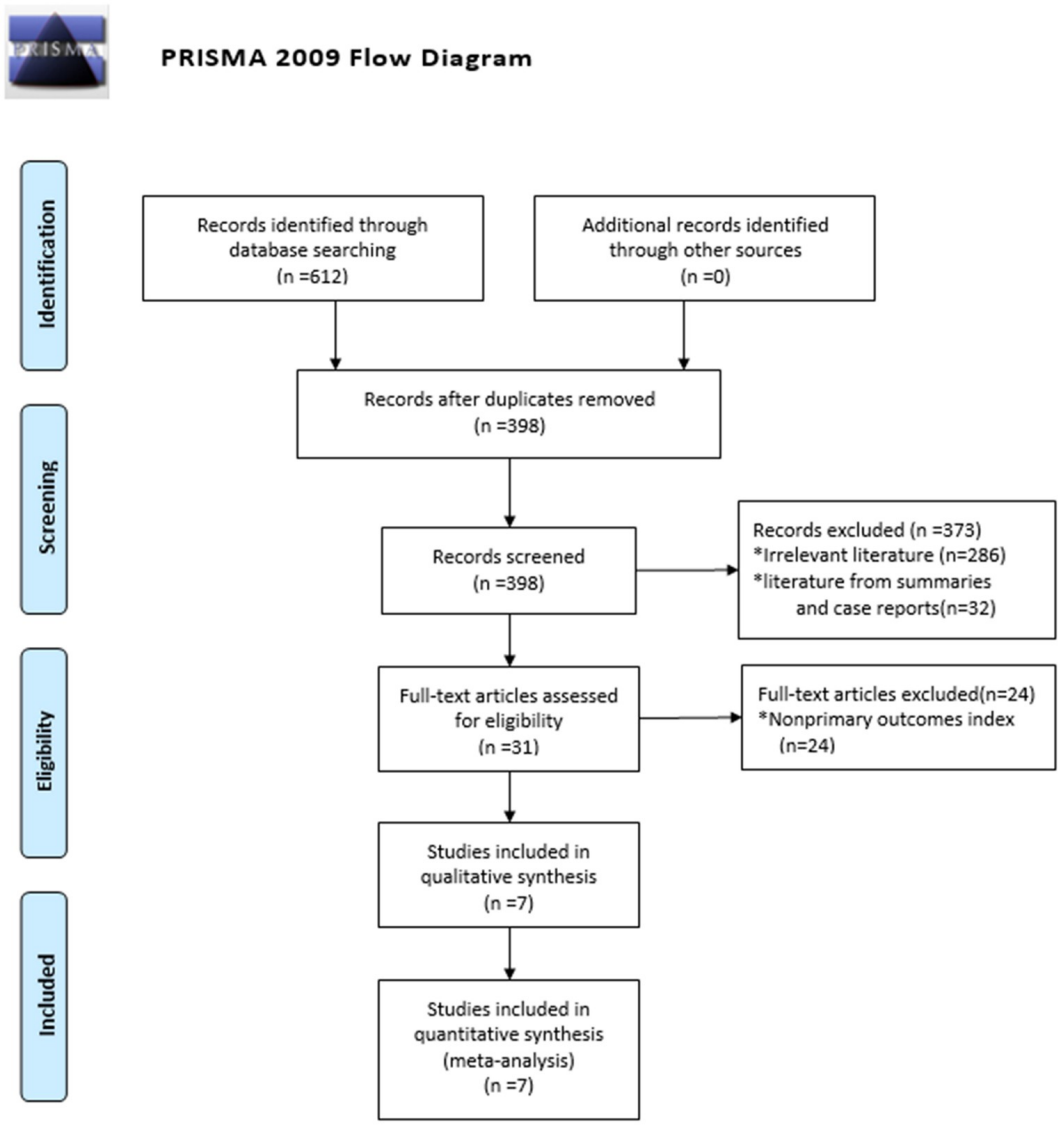
The literature screening process and results. *From*: Moher D, Liberate A, Tetzlaff J, Altman DG. The PRISMA Group (2009). *P*referred *R*eporting *i*tems for Systematic Reviews and *A*nalyses: The PRISMA Statement. PLoS Med 6(7): e1000097. doi: 10.1371/journal.pmed1000097. **For more information, visit** www.prisma-statement.org.

Search words were: ‘botulinum toxin’, ‘botulin’, ‘botulinum neurotoxin’, ‘botulinum’, ‘botulinum toxin’, ‘botulinum neurotoxins’, ‘botulism toxin’, ‘botulin toxin’, ‘botulinum toxin’, ‘botulinum a toxin’, ‘sacral neuromodulation’, ‘sacral nerve electrostimulation’, ‘sacral nerve stimulation’, ‘OAB’, ‘overactive bladder’, and ‘urinary bladder hyperactivity’. A comprehensive literature search was carried out on November 11, 2019 using PubMed, Embase, and Cochrane Library databases. Simultaneously, we looked up relevant studies by referencing other literature. The literature publication dates were limited to the period between July 2002 to November 2019. The study selection process is summarized in Fig 1.

### Literature screening and data extraction

Two researchers independently screened the literature and extracted the data. They cross-checked the data, and if they encountered differences of opinion, they discussed with each other to resolve disputes of opinion or submitted the issue to a third party for resolution. A self-made data extraction chart was adopted to extract the following data: ① the basic information included in the study, including research objective, authors, the publication where the literature was published and the time of publication, and others; ② the baseline characteristics of the research participants, including each group’s average age and gender distribution; ③ the primary factors of bias risk assessment; ④ all relevant outcome indicators and data related to results measurement.

### Bias risk assessment for research inclusion

The quality of the nonrandomized studies was assessed using NOS with some modifications to match this study’s needs. The NOS included observational studies, specifically including patient selection, comparability and results assessments of the research participants, with the highest score at 9 points (Table 1).

**Table 1:** Check list for quality assessment and scoring of nonrandomized studies.

Check List
Selection 1. Assignment for treatment: any criteria reported? (if yes, one star) 2. How representative was the control group in comparison with the refractory overactive bladder patients? (if yes, one star; no star if the patients were selected or selection of group was not described) 3. How representative was the treatment group in comparison with the refractory overactive bladder patients? (if drawn from the same community as the reference group, one star; no star if drawn from a different source or selection of group was not described) Comparability 4. Group comparable for 1, 2, 3, 4 (if yes, two stars; one star was assigned if one of these five characteristics was not reported even if there were no other differences between the two groups and other characteristics had been controlled for; no star was assigned if the two groups differed) 5. Group comparable for 5, 6, 7 (if yes, two stars; one star was assigned if one of these four characteristics was not reported even if there were no other differences between the two groups and other characteristics had been controlled for; no star was assigned if the two groups differed) Outcome assessment 6. Clearly defined outcome of interest (yes, one star for information ascertained by record lincage or interview; no star if this information was not reported) 7. Adequacy of follow-up (one star if follow-up 90%)

Comparability variables: 1 age; 2 gender; 3 voiding diary; 4 quality of life; 5 BTX treatments dose; 6 mean prior BTX treatments; 7 mean duration of last BTX to SNM.

### Statistical analysis

Stata 12.0 software was used to conduct a meta-analysis of the data. Publication bias was estimated based on the funnel plot. Effect size was expressed as point estimation value and 95% confidence interval (CI). Studies with an I^2^ statistic>50% or *p*< 0.1 were considered to have significant heterogeneity. A fixed-effects model was used if there was no significant heterogeneity. Otherwise, random-effects model were employed in the meta-analysis.

## Results

An initial search produced 612 relevant articles, and after strict screening for inclusion and exclusion criteria. Eventually, seven retrospective studies[6–12] were involved in this study. The literature screening process and results are shown in Fig 1. The seven included retrospective studies included 319 patient cases. The baseline characteristics and NOS score results for research inclusion are illustrated in Table 2.

**Table 2:** The baseline characteristics and NOS score results for research inclusion.

Study	Year	Origin country/period	Population	Age	BTX treatments (dose(units)	Mean prior BTX treatments	Mean duration of last BTX to SNM	Sample content	Outcome	Follow-up time	NOS score
Smits, M. A et al.	2013	The Netherlands (2005–2010)	refractory idiopathic OAB(16/20 Female (80%)	56years (range 37–82)	100–300	range 1–4	23months (range 7-53).	20	success rate: 14/20 70%	23 months (7–53 months)	6
Abtahi, B et al.	2013	United Kingdom (2007–2012)	refractory detrusor overactivity (DO)	48years (range 34–62)	200(100–300)	range 1–4	NA	24	success rate: 13/24 54.16%	6 weeks, 6 months, 12 months	7
Hoag, N et al.	2016	Australia (2010–2015)	refractory idiopathic OAB(77/83 Female (92.8%)	60.9years (range 22–86)	100–300	2.83 (range 1–13)	11.8months (range 3–30)	36/47	success rate: 23/36 (63.9%); 33/47 (70.2%).	29.1 months (at least 12 months)	7
Morton, H et al.	2015	United Kingdom (2011–2014)	refractory OAB	52years (range 38–69)	200(100–300)	1.6(range 1–5)	22.2months (range 6.3–63.2)	36/26	success rate: 23/36 (63.9%); 16/26 (61.54%).	NA	6
Forrest, A et al.	2011	United Kingdom (NA)	refractory OAB(23/23 Female 100%)	45years (range 32–63)	200(100–300)	1.4(range 1–4)	NA	11/12	success rate: 2/11 (18%); 6/12 (50%).	NA	5
Singh, R et al.	2016	United States (2002–2013)	refractory OAB(75/75 Female 100%)	64.3years (range 51–73)	100–300	range 1–6	NA	8/67	success rate: 6/8 (75%); 42/67 (62.69%)	17.8–40.5 months	7
Agarwal, S et al	2018	United States (2007–2017)	refractory OAB(29/32Female 91%)	69years (range 52–76)	100–300	range 1–4	14months	32	success rate: 20/32 63%	34 months	7

### Meta-analysis results

**Fig 2:**
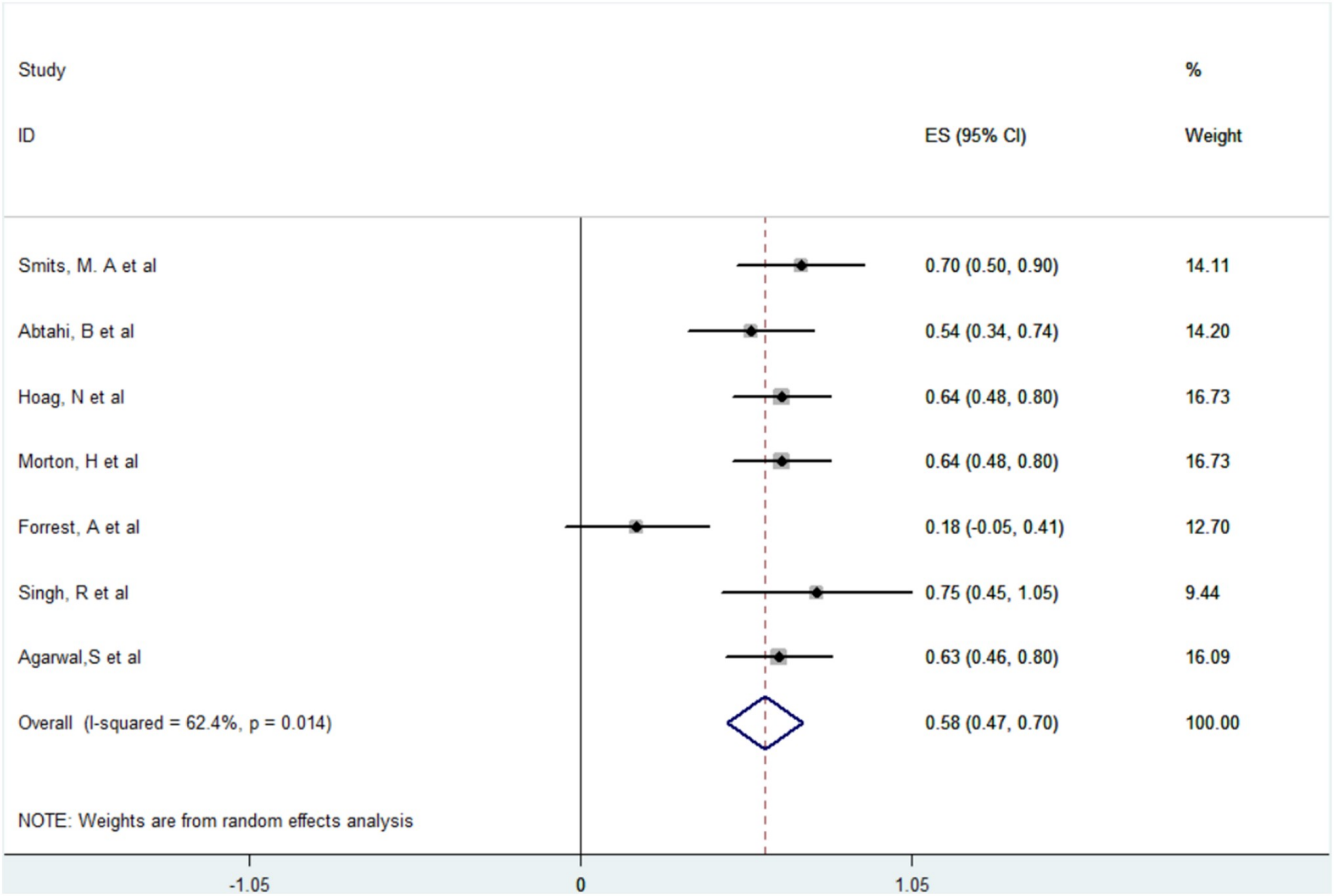
A meta-analysis of the success rate of refractory OAB patients who used SNM therapy after ending BTX therapy.

The success rate of refractory OAB patients choosing SNM as replacement therapy after ending BTX therapy. The results of meta-analysis are shown in Fig 2. There was a total of six included studies[6–12] tested for heterogeneity: each index where there was significant heterogeneity (*p* = 0.014, I^2^ = 62.4%). A randomized effects model was adopted to conduct a synthesized analysis of the data. We used a fixed effects model to synthesize the size of each index’s group mean. The overall results and those of the random effects model were similar, demonstrating that the synthesized outcomes had good stability and were reliable. Synthesis of the effects size was as follows: After the six included studies were weighted and synthesized, the success rate of refractory OAB patients who used SNM therapy after ending BTX therapy was 58.5%, 95% CI (0.47–0.70).

**Fig 3:**
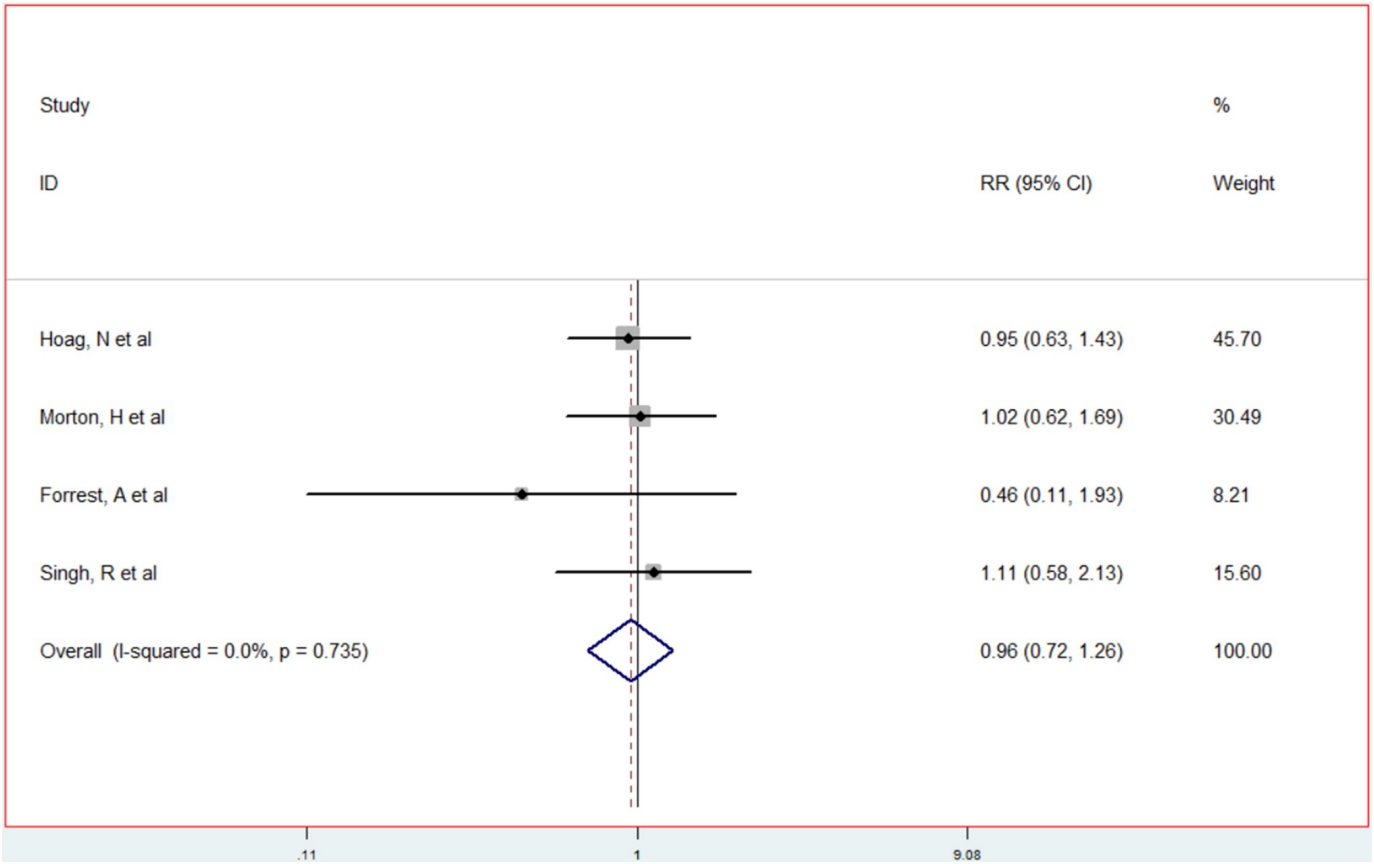
Meta-analysis of the efficacy comparison between refractory OAB patients who chose SNM therapy after ending BTX therapy and the initial choice of SNM therapy.

We compared outcomes in patients with refractory OAB who chose SNM therapy after ending BTX therapy and those for whom the initial choice was SNM therapy. The results of meta-analysis are shown in Fig 3. There was a total of four included studies[6–9] tested of heterogeneity: For each index, the magnitude of heterogeneity was zero (*p* > 0.10, I^2^ = 0); therefore, a fixed effects model was adopted to conduct a synthesized analysis of the data. Synthesis of the effect size: After the four included studies were weighted and combined, the resulting statistic RR was 0.96, [95% CI: 0.72–1.26], and there was no significant difference between the two groups (*p* = 0.735).

**Fig 4:**
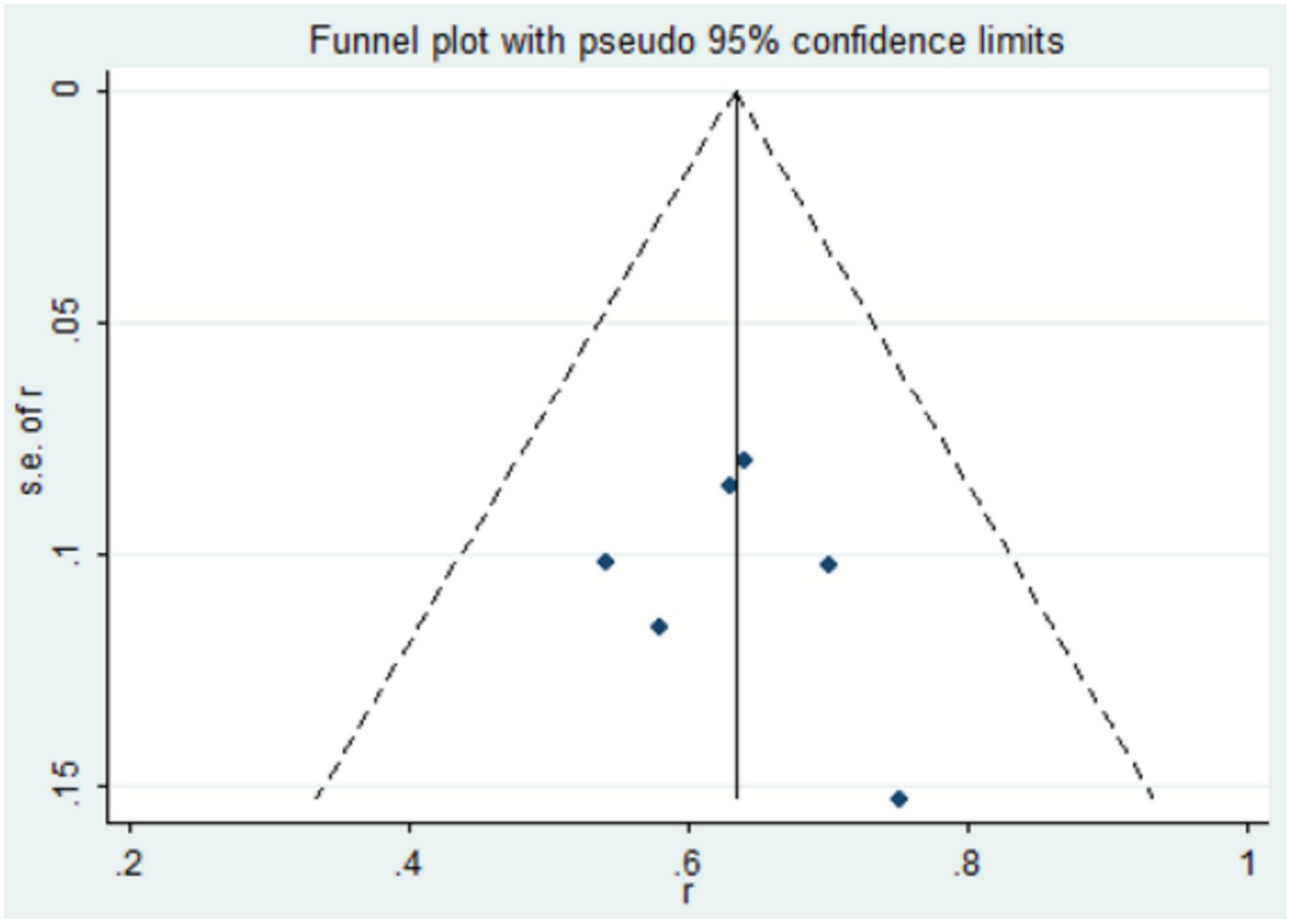
Funnel plot assessing potential publication bias.

Publication bias is defined as the problem that results from systematic differences between the results of all the completed studies on a topic and the results of the subset of those studies that are published[13]. The plot in Fig 4 resembles a symmetrical inverted funnel (95% CI), inside which are all studies included in our meta-analysis. There was no evidence of publication bias as revealed by the distribution of the funnel plot.

## Discussion

The American Urological Association (AUA) and the Society of Urodynamics, Female Pelvic Medicine & Urogenital Reconstruction (SUFU)’s updated guidelines classify BTX and SNM as third-line treatment options for refractory OAB[14]. Henriet et al.[15] performed a systematic retrospective study and found that injecting BTX inside the detrusor muscle effectively increased bladder capacity, improved OAB symptoms, and enhanced QOL. Nevertheless, the most commonly seen side-effect was incomplete emptying and intermittent urinary catheterization with accompanying increased risk of urinary tract infections. After undergoing BTX therapy, the possibility that refractory OAB patients may require insertion of an intermittent urinary catheter varied from 16% to 42%[16,17]. For patients in whom BTX therapy for refractory OAB failed or the patients appeared unwilling to continue receiving BTX therapy, SNM was the only minimally invasive replacement therapy method.

Our meta-analysis revealed that the success rate of SNM therapy after the initial choice and failure of BTX therapy was 58.5%, 95% CI (0.47–0.70). Furthermore, there was no significant difference in terms of the effectiveness of SNM therapy after the initial choice and failure of BTX therapy as compared to the initial choice of SNM therapy, with an RR of 0.96, [95% CI: 0.72–1.26] (not significant, *p* = 0.735). This may be related to the differences created between the preceding research results and the action of BTX and SNM on the mechanism of action of OAB. BTX quickly binds the neuromuscular end-plate to inhibit the release of acetylcholine from presynaptic membranes, thereby removing innervation in the form of muscle relaxation and paralysis. It also has the therapeutic effect of alleviating spasms and stiffness. It is also related to the mechanism of SNM therapy in treatment of OAB and adjusting micturition reflexes with sacral cord afferent impulses. Compared to the local response to BTX, SNM has an even more central role.

In terms of treating OAB, SNM therapy has long-term and stable healing effects; its most commonly seen side-effect is regional pain at the implant site, with an incidence of 3–42%. Its side-effects include impaired defecation (4–7%), infection (4–10%), and electrode malposition (1–21%)[18,19]. Among refractory OAB patients who initially chose SNM therapy, there were some who abandoned SNM therapy because it was ineffective or they could no longer tolerate the side-effects. There is currently a major controversy regarding whether they could continue to use BTX as second line therapy for SNM therapy. Kirkpatrick et al.[20] performed a retrospective analysis and found that when patients with urge incontinence OAB received BTX therapy after failure of SNM therapy, it was even more effective than was BTX therapy. By contrast, recent studies reported that the success rate when OAB patients chose BTX therapy after the initial choice and ineffectiveness of SNM therapy was only 27%–39%[21,22]. Currently, there is a need for more high-quality research before one can confirm the efficacy of OAB in those who receive BTX therapy after the failure of initially undergoing SNM therapy.

This research’s advantages are demonstrated in the wide-ranging literature retrieval, and the included standard can be reproducibly operated. The methodology is feasible, and the assessment and data collection are reproducible. Our research is the first systematic review with meta-analysis assessing refractory overactive bladder patients who chose sacral neuromodulation therapy after failed onabotulinumtoxinA treatment, which guides clinical practice and future research. There were several limitations of this study: 1) there was a limited amount of pertinent research, and most of the research samples were relatively small and there is a paucity of randomized controlled trials; 2) despite the fact that we selected relatively better examples of recently published literature, there remained problems with homogeneity and sensitivity. As a result, there is a need for more comprehensive studies with acceptable homogeneity and sensitivity; 3) there were also linguistic limitations. We included only English publications; therefore, we might have missed pertinent research published in other languages. Large scale and multi-center studies are needed to further validate our results.

## Conclusion

Current evidence suggests that patients with refractory OAB who either failed or were dissatisfied with BTX therapy can chose SNM therapy. It is effective and shows no substantial differences in terms of outcomes from SNM initial therapy.

## Supporting information

S1 Checklist PRISMA 2009 checklist. (DOC)
